# New Approach to Increasing Rice Lodging Resistance and Biomass Yield Through the Use of High Gibberellin Producing Varieties

**DOI:** 10.1371/journal.pone.0086870

**Published:** 2014-02-19

**Authors:** Ayako Okuno, Ko Hirano, Kenji Asano, Wakana Takase, Reiko Masuda, Yoichi Morinaka, Miyako Ueguchi-Tanaka, Hidemi Kitano, Makoto Matsuoka

**Affiliations:** Bioscience and Biotechnology Center, Nagoya University, Nagoya, Aichi, Japan; China Agricultural University, China

## Abstract

Traditional breeding for high-yielding rice has been dependent on the widespread use of fertilizers and the cultivation of gibberellin (GA)-deficient semi-dwarf varieties. The use of semi-dwarf plants facilitates high grain yield since these varieties possess high levels of lodging resistance, and thus could support the high grain weight. Although this approach has been successful in increasing grain yield, it is desirable to further improve grain production and also to breed for high biomass. In this study, we re-examined the effect of GA on rice lodging resistance and biomass yield using several GA-deficient mutants (e.g. having defects in the biosynthesis or perception of GA), and high-GA producing line or mutant. GA-deficient mutants displayed improved bending-type lodging resistance due to their short stature; however they showed reduced breaking-type lodging resistance and reduced total biomass. In plants producing high amounts of GA, the bending-type lodging resistance was inferior to the original cultivars. The breaking-type lodging resistance was improved due to increased lignin accumulation and/or larger culm diameters. Further, these lines had an increase in total biomass weight. These results show that the use of rice cultivars producing high levels of GA would be a novel approach to create higher lodging resistance and biomass.

## Introduction

Since the beginning of cereal cultivation, a continuous challenge has been to increase grain yield through breeding. One example is the introduction of dwarfism into wheat and rice, which led to a remarkable success in increasing grain production in the 1960s, consequently averting a predicted large-scale famine. This achievement was referred to as the “Green Revolution” [1]–[3]. One explanation for high grain production during the Green Revolution was the combination of widespread fertilizer use and the cultivation of high-yielding, semi-dwarf varieties. The availability of sufficient fertilizer is essential to increased grain production, but also promotes stem and leaf elongation, resulting in an increase in total plant height. Traditional varieties of wheat and rice grew excessively tall when fertilizer was abundant and became susceptible to lodging, resulting in significant yield loss. In contrast, varieties with short stature are resistant to lodging even when fertilized excessively, and thus are capable of supporting their own body even if high grain yielding trait is introduced.

The genes responsible for imparting short stature to rice and wheat used in the Green Revolution have been identified by Sasaki et al. [4] and Peng et al. [5], respectively. Interestingly, both genes are involved in the synthesis or signaling of gibberellin (GA). For example, rice *sd-1* mutants have loss-of-function mutations in the GA synthesis gene, *GA 20 oxidase2 (semi dwarf-1; SD-1)*. On the other hand, wheat *Reduced height-1* (*Rht-1*) mutants have gain-of-function mutations in a gene encoding a suppressor of a GA signal known as the DELLA protein. It is noteworthy that the genes selected for in rice and wheat dwarfism depend on GA, despite the fact that mutations in genes for other phytohormones (e.g. strigolactone, brassinosteroid, and auxin) also can result in dwarfism. Furthermore, Asano et al. [6] reported that seven different *sd-1* alleles have been independently selected for reducing rice height in the Philippines, Japan, the United States, and China. This clearly demonstrates the importance of *sd-1* mutations in modern rice breeding.

As mentioned above, efforts to increase grain production are ongoing, although a substantial increase has been slow to come after the Green Revolution [5]. To avoid future food crises arising from climate change and the rising global population, it is vital to further improve grain production using alternative strategies. Furthermore, it is important to note that cereal crops have recently been used as alternative energy sources, and thus it is now necessary to breed plants for potential biofuels. In terms of biomass, dwarfism would be considered an unfavorable trait. Conversely, GA-overproduction or hypersensitivity causes taller plant height and increases total biomass production, although it may decrease lodging resistance.

In addition to plant height, lodging resistance also depends on the physical strength of culms [7]. However, few reports exist describing the effects of GA on culm characteristics. To address this, we examined several parameters which directly or indirectly affect features of the culm and lodging resistance by using eight rice mutants/lines with a dwarf or tall phenotype. Through the investigations, we found that GA has positive effects on culm traits and both positive and negative effects on lodging resistance. We propose an alternative breeding strategy to create rice with high lodging resistance and high biomass via GA.

## Materials and Methods

### Plant materials and cultivation

For the *sd-1* mutants, three isogenic lines; two intermediate alleles (*sc^Reimei^* and *sc^Shiranui^*), and one null allele (*sc^TN-1^*) were used with the recurrent parent, Norin 29 [8]–[10]. For the ent-kaurene oxidase (KO) mutant, *d35*/*Tan-Ginbozu* was used with its original cultivar Ginbozu [11]. *gid1-8* and *Slr1-d1* were used as GA-signaling mutants with their original cultivar Taichung 65 (T65) [12], [13]. For tall plants, *SD-1^K^*, which was obtained from National Institute of Agrobiological Sciences [14], and *elongated uppermost internode 1* (*eui1*) [15], [16] were used with their original cultivar Nipponbare. Field experiments for the phenotyping of agronomical traits were conducted at the Togo Field Science and Education Center of Nagoya University. Seeds were sown in nursery boxes and grown to the fifth-leaf stage. Subsequently, seedlings were transplanted to a paddy field with a spacing of 15×30 cm in a 1 m^2^ area. Plants grown at the perimeter of the field were not included in the analyses.

### Morphological traits, culm diameter, above-ground biomass, and total grain yield

The following measurements were conducted using each cultivar or mutant line at the near full-ripening stage (40 d after heading). To assess culm diameter, the central section of fresh culms were hand-sectioned, and the outer diameter of the major axis was measured. For culm and panicle length, tiller number, above-ground biomass, and grain yield, plants were air-dried for 2 weeks before measurement. Above-ground biomass was measured after excluding the panicles and the roots, and grain yield were measured by the total grain weight per plant.

### Measurement of physical properties relevant to lodging resistance

The lodging resistance factor (cLr), a parameter to evaluate resistance to bending-type lodging was determined using the method of Grafius and Brown [17]. To assess the mechanical strength of culms, five culms were sampled from each plant at near full-ripening stage (40 d after heading). Bending load at breaking was measured at a distance of 4 cm from the supporting point by using the load-testing machine, Tensilon RTM-25 Orientic, according to the method described by Ookawa and Ishihara [18]. Physical parameters were calculated using the following formula: M =  section modulus × maximum bending stress [19]. M is the bending moment of the internode at breaking (g • cm). Section modulus (SM)  = π/32× (a_1_^3^b_1_ − a_2_^3^b_2_)/a_1_, where, a_1_ is the outer diameter of the minor axis in an oval cross-section, b_1_ is the outer diameter of the major axis in an oval cross-section, a_2_ is the inner diameter of the minor axis in an oval cross-section and b_2_ is the inner diameter of the major axis in an oval cross-section.

### Lignin measurement

The amount of lignin in the uppermost culm was determined using the method of Suzuki et al. [20], which specifically measures thioglycolic acid lignin.

### RNA isolation and quantitative RT-PCR analysis

Total RNA was prepared from uppermost internodes, as described [21]. First strand cDNA was synthesized from total RNA using an Omniscript Reverse Transcription Kit (Qiagen). Quantitative real-time RT-PCR (qRT-PCR) was performed with the LightCycler System (Roche, Basel, Switzerland) and the SYBR Green PCR Kit (Qiagen). The results were confirmed using more than three independent biological replicates. The ubiquitin gene from rice was used as an internal standard for normalizing cDNA concentration variations.

## Results

### Relationship between plant height and lodging resistance

**Figure 1:**
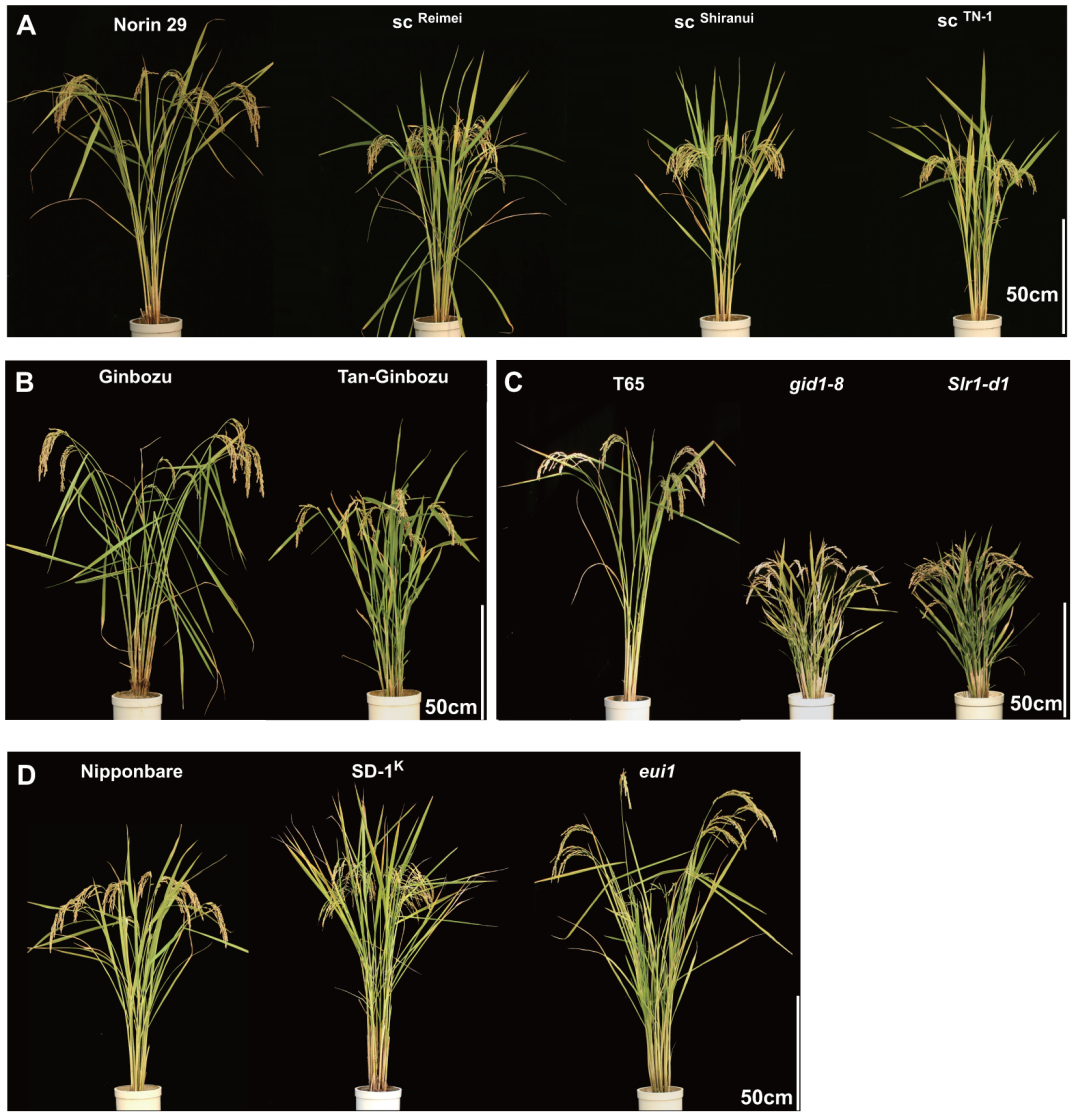
Gross morphology of plants used in this study. (A) to (D) Photographs show GA-related mutant lines 40 days after heading and are compared with original lines (Norin 29, Ginbozu, T65, and Nipponbare) at far left of each panel.

To evaluate how GA impacts plant traits related to culm features and lodging resistance, eight GA-related mutants/lines with semi-dwarf, dwarf, or tall phenotypes were investigated (Fig. 1). For semi-dwarf plants, we selected two kinds of mutants defective in GA biosynthesis; both have been used as commercial cultivars. One group contained different mutant alleles of *sd-1*; SD-1 is an enzyme responsible for the late step in GA biosynthesis, and thus *sd-1* mutants have reduced levels of bioactive GA and a dwarf phenotype. Three isogenic lines; two *sd-1* intermediate alleles (*sc^Reimei^* and *sc^Shiranui^*), and one *sd-1* null allele (*sc^TN-1^*) were compared to their recurrent parent, Norin 29 (Fig. 1A) [8]–[10]. Among the three, *sc^Reimei^* is the weakest allele, whereas, *sc^TN-1^* is the most severe. Another GA biosynthesis mutant, *d35*/*Tan-Ginbozu*, contains an mild mutation in the gene for ent-kaurene oxidase (KO), which encodes an enzyme for an early step in GA biosynthesis (Fig. 1B, [11]). It is noteworthy that *Tan-Ginbozu* was named the most productive cultivar in Japan in 1951.

We also used two GA-insensitive dwarf mutants (Fig. 1C). *gid1-8* is a mutant partially defective in the GA receptor, GIBBERELLIN INSENSITIVE DWARF 1 (GID1), and shows reduced GA-binding activity [13]. *Slr1-d1* expresses an activated form of a suppressor protein for GA signaling, which results in repression of GA signaling and the inhibition of plant growth [12]. This type of mutation is inherited as a dominant trait, and the *RHT-1* mutants used in the “wheat Green Revolution” depend on the same mechanism as rice *Slr1-d1* lines [5]. For GA-related tall lines, we used *SD-1^K^* and *elongated uppermost internode 1 (eui1)* (Fig. 1D). *SD-1^K^* was produced from japonica cultivar Nipponbare by introgression with the *SD-1* locus of an indica cultivar, Kasalath [14]. This introgressed plant grows taller than the original line, Nipponbare, largely because the *SD-1* allele of Kasalath, *SD-1^K^*, has higher enzymatic activity than Nipponbare [22]. The *eui1* mutant is defective in the gene encoding the GA-inactivating enzyme, CYP450 monooxygenase (CYP714D1) and exhibits an elongated internode phenotype [15], [16].

**Figure 2:**
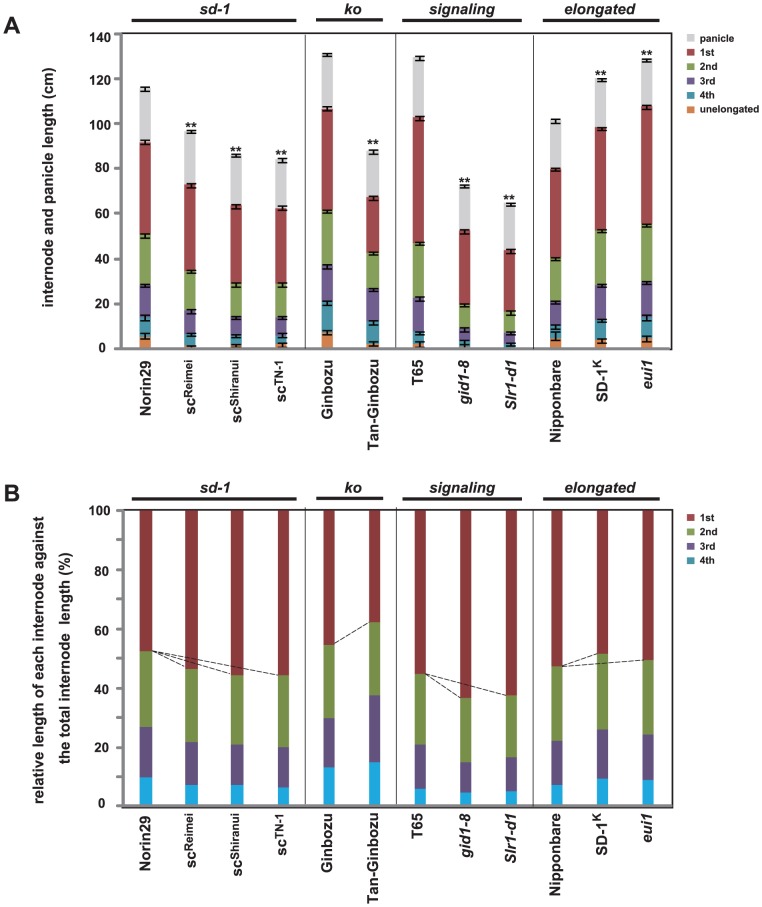
Culm length of rice lines used in this study. (A) Diagram of plant height and internode length. (B) Elongation pattern of the culms. The length of the upper four internodes were averaged in each plant line, and the relative length against the total internode length is shown (n = 5). Original lines are shown at the left of each group. Mutant lines defective in GA signaling, and plants with elevated GA levels are classified into a single group. Error bars indicate the standard error. Asterisks indicate statistically significant differences with respect to original cultivars (*P*<0.01; Dunnett [33]).

In Fig. 2, we compare the total culm length of these GA-related mutants. The six semi-dwarf or dwarf mutants showed differential levels of dwarfism ranging from 43–79% relative to the original cultivar, whereas mutant/line with taller phenotypes showed a 23–34% increase in culm length. We also observed a relative elongation pattern for each internode (Fig. 2B). The internode elongation pattern was similar between three *sd-1* and two GA-signaling mutants; in other words, compared to their original cultivars, the length of the lower internodes (3^rd^–4^th^) was decreased more than that of the uppermost (1^st^) internode. Conversely, the length of the uppermost internode was decreased more than that of the lower internodes (3^rd^–4^th^) in *Tan-Ginbozu* when compared with Ginbozu. *SD-1^K^* showed a contrasting pattern to the *sd-1* mutants and the lower internodes (3^rd^–4^th^) increased more than that of the lower internodes, whereas the internode elongation pattern of *eui1* was similar to the original cultivar Nipponbare.

**Figure 3:**
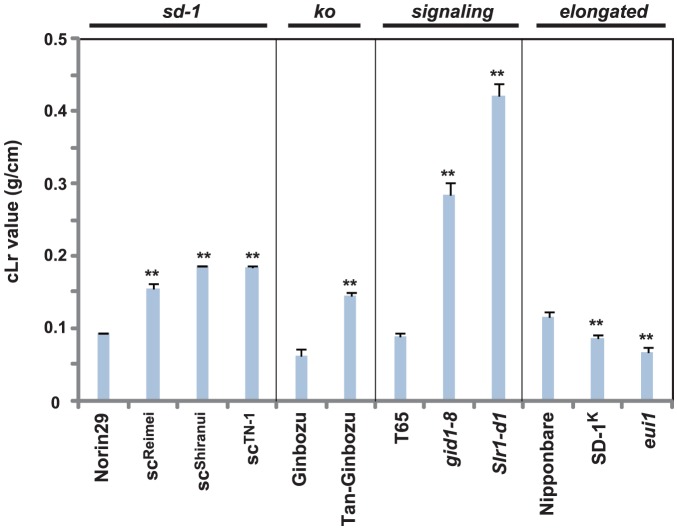
Bending-type lodging resistance of plant lines enhanced or suppressed for GA signaling. Mutants suppressed for GA signaling show improved cLr values, whereas GA-enhanced plants show reduced cLr values (n = 5). The asterisks indicate statistically significant differences in comparison to the original line (*P*<0.01; Dunnett [33]).

As mentioned above, dwarfism is a preferable trait in terms of lodging resistance. We directly examined the lodging resistance parameter (cLr), which is a factor for evaluating resistance to bending force. As expected, *sd-1* mutants showed a 1.7 to 2.0-fold and *Tan-Ginbozu* a 2.3-fold increase in cLr values compared to the original cultivars (Fig. 3). Mutant lines exhibiting more severe dwarfism, e.g. *gid1-8* and *Slr1-d1*, showed higher levels of lodging resistance relative to the original strain (3.2 and 4.7-fold higher, respectively). In contrast, tall mutants showed decreased stem bending resistance (e.g. 0.6 to 0.8-fold lower than Nipponbare). These clearly demonstrate that dwarfism promotes bending-type lodging resistance. This is largely because dwarfism results in a lower gravity point and decreases the fresh weight of plants, which consequently reduces the “self-weight” moment of the aerial part of the plant [23].

### Culm structure, tiller number, and total biomass of GA-related mutants

**Figure 4:**
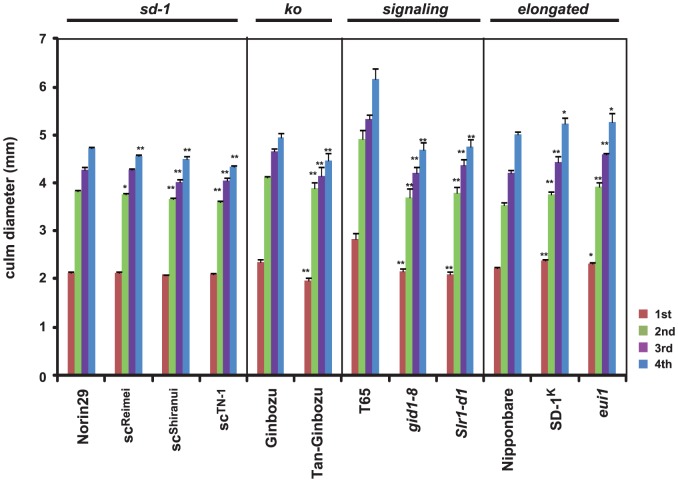
Enhancement and suppression of GA signaling leads to increased and reduced culm diameter, respectively. Culm diameters of uppermost to the fourth internode were measured (n = 5). The asterisks indicate statistically significant differences with respect to the original line (*P<0.05, **P<0.01; Dunnett [33]).

Although plant height influences lodging resistance, the characteristics of the culm are also important for lodging resistance. The physical strength of culms is primarily determined by morphology and the quality of culm material [7]. When culm diameter was measured, *sd-1* mutants showed significantly reduced culm diameter in the lower internodes, whereas more severe dwarf mutants such as *Tan-Ginbozu* and the GA-signaling mutants showed a strong reduction in culm diameter at all internodes (Fig. 4). At the first internode, *sd-1* mutants showed a 0–2.0% reduction, *Tan-Ginbozu* exhibited a 15.4% reduction, and *gid1-8* and *Slr1-d1* showed a 23.0 and 25.5% reduction compared to their original cultivars, respectively. In contrast, the taller GA mutants possessed increased culm diameter at all internodes, with the uppermost internode of SD-1^K^ or *eui1* showing an increased diameter 7.0% or 3.5% relative to the original strain, respectively. Thus these results demonstrate that there is a positive correlation between GA and culm diameter in rice.

**Figure 5:**
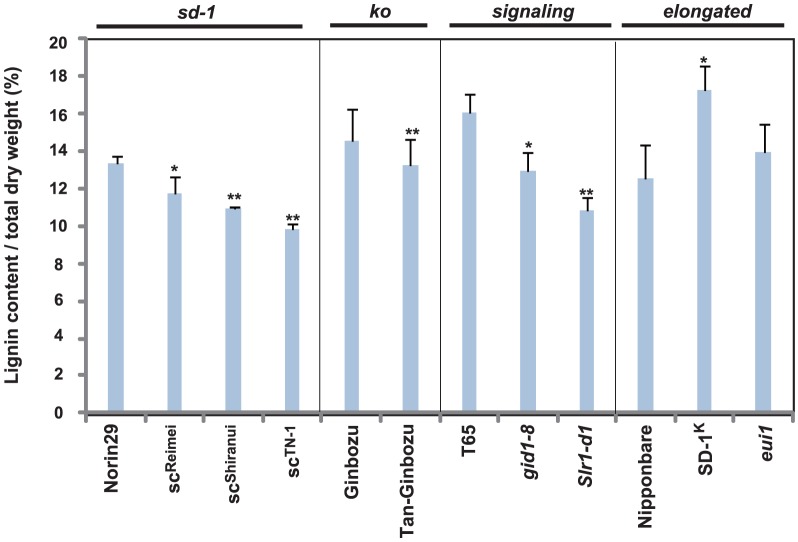
Lignin content of plants used in this study. Lignin was measured by thioglycolic acid method at the uppermost internode (Suzuki *et al*., 2009) and divided by the total dry weight of the sample (n = 3). The asterisks indicate statistically significant differences with respect to the original cultivar (*P<0.05, **P<0.01; Dunnett [33]).

**Figure 6:**
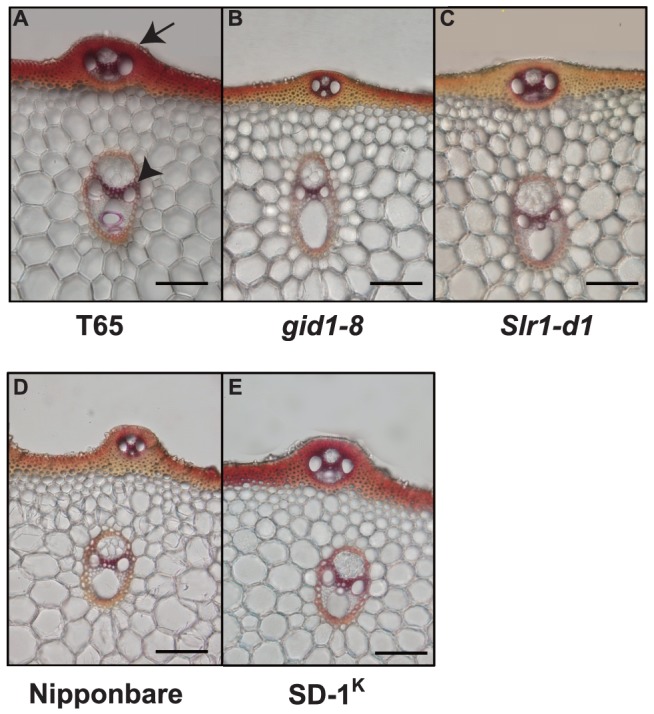
Phloroglucinol staining of transverse sections at the second internode of various rice cultivars and mutants. Compared to the original lines, *gid1-8* and *Slr1-d1* show weak staining and SD-1^K^ shows stronger staining, especially in the sclerenchyma layer. The arrow and arrowhead show sclerenchyma and a vascular bundle, respectively. Bar  = 100 µM.

**Figure 7:**
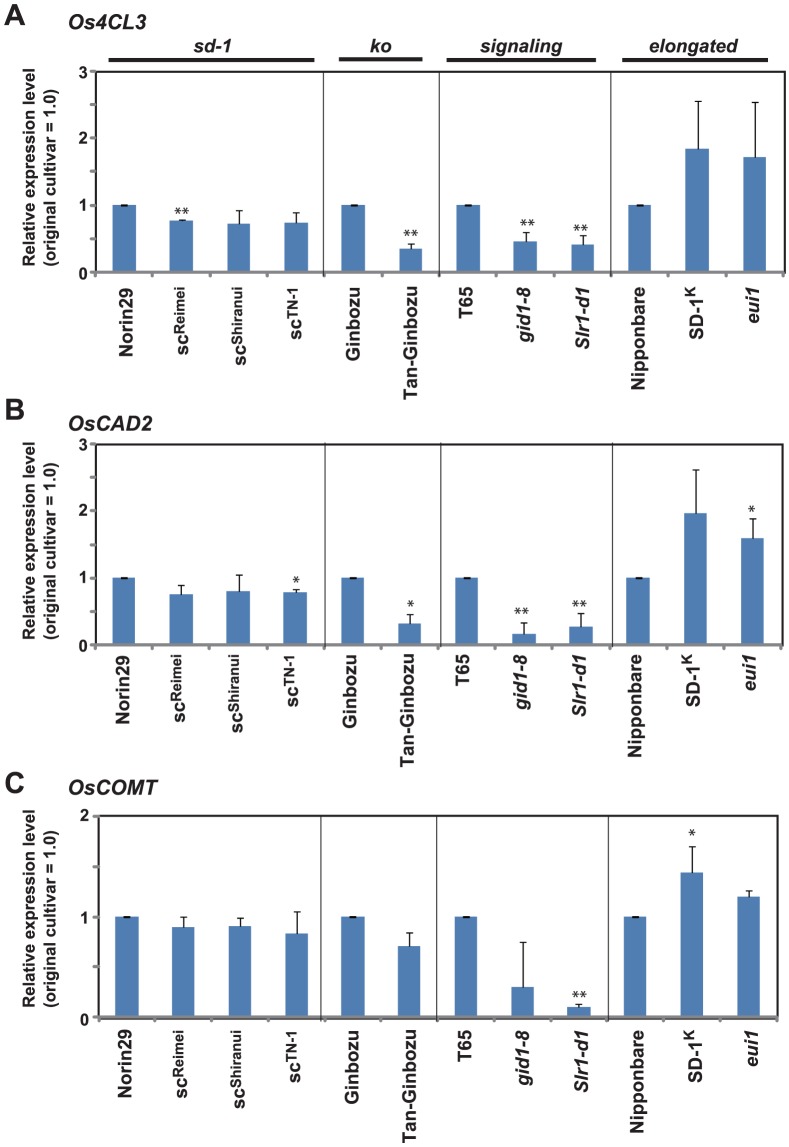
Expression of rice lignin biosynthesis genes. The uppermost internode of each plant at 13 days after flowering was used for analysis (n< = 3). Expression was analyzed by the quantitative reverse transcription PCR. (A) Expression of *Os4CL3* gene. (B) expression of *OsCAD2* gene. (C) Expression of *OsCOMT* gene. Asterisks indicate statistically significant differences relative to the original lines (*P<0.05, **P<0.01; Dunnett [33]).

To evaluate the physical quality of culm material, we measured the lignin content of the uppermost internode of each plant (Fig. 5), because higher levels of lignin cause enhanced physical strength of the culm material [7]. All dwarf mutants showed a decrease in lignin content compared to their original cultivar. In the case of tall GA mutants, *SD-1^K^* showed a significant increase in lignin accumulation, whereas *eui1* showed an increase that was not significant. We also investigated what part of the internode was affected in lignin accumulation by staining sections of the T65 and Nipponbare lines with phloroglucinol (Fig 6). Strong staining was observed primarily in the sclerenchyma with some staining in the vascular bundles; these are the two most important tissues for determining the physical quality of the culm (see arrow and arrowheads in Fig. 6A, respectively). The staining of *gid1-8* and *Slr1-d1* was less intense than the original lines, whereas *SD-1^K^* stained more strongly than its parent. For the other mutants, differences were not observed relative to the original line (data not shown). We further analyzed the expression of lignin biosynthesis genes at the uppermost internode of each plant at 13 days after flowering where secondary cell wall is extensively synthesizing [24]. The expression of *Os4CL3*, *OsCAD2*, and *OsCOMT* genes were analyzed, since they are actually (*Os4CL3* and *OsCAD2*) or presumed (*OsCOMT*) to be involved in rice lignin formation [25], [26], [27]. For the *sd-1* mutants, compared to Norin 29, they showed slight decreased expression in all the lignin biosynthesis genes examined (Figure 7), but most of the decreases were not significant, possibly reflecting the small amount of reduction in their lignin content compared to Norin 29 (Figure 5). In contrast, Tanginbozu, *gid1-8*, and *Sl1-d1* mostly led to significant decreased lignin gene expression compared to their original cultivars. For the high GA-producing rice, SD-1^K^ and *eui1*, although *OsCAD2* in *eui1* and *OsCOMT* in SD-1^K^ was the only genes significantly up-regulated compared to Nipponbare, both plants showed increase in the expression of all the lignin biosynthesis genes examined. Altogether, these results support that GA induces lignin accumulation in rice.

**Figure 8:**
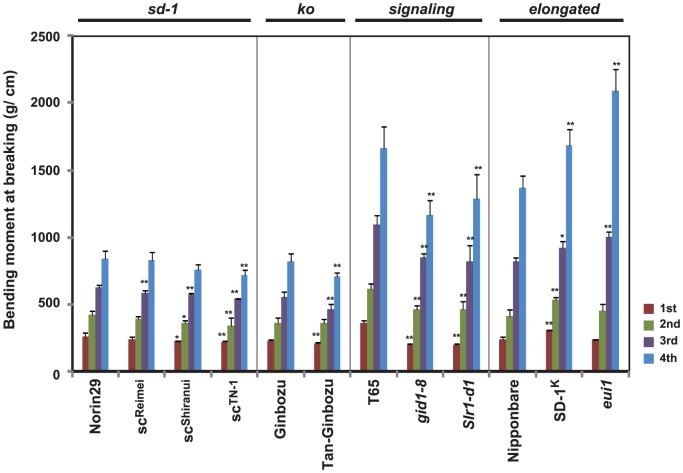
Bending moment at breaking lodging. The region apical to the fourth internode of each plant was used for analysis (n = 5). Asterisks indicate statistically significant differences relative to the original lines (*P<0.05, **P<0.01; Dunnett [33]).

Next, we measured the force of bending moment at the breaking point of the culm, a parameter that evaluates the physical strength of the culm. All semi-dwarf and dwarf lines showed reduced bending moment values relative to the original cultivars (Fig. 8). For example, *gid1-8* and *Slr1-d1* showed 22–44 and 23–44% reduction in all internodes, respectively. In contrast, the tall plants, *SD-1^K^* and *eui1*, showed 1.1–1.3 and 1.0–1.5-fold higher values relative to the original line, respectively. These results clearly demonstrate that GA increases the lignin content and/or culm diameter, and results in enhanced physical strength of the culm.

**Figure 9:**
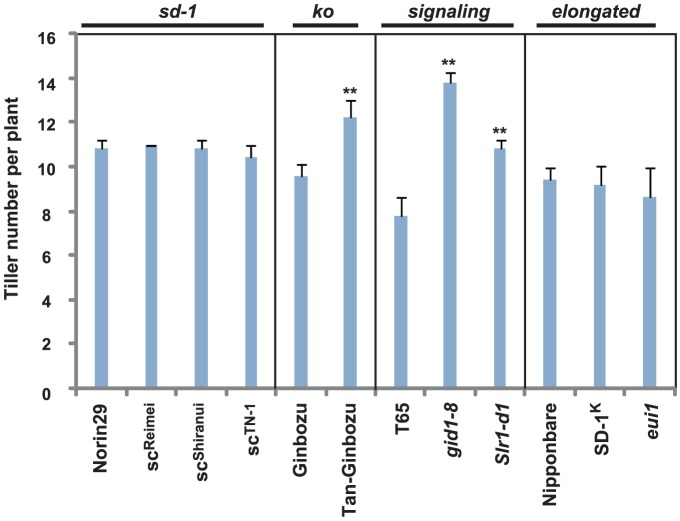
Tiller numbers (n = 5) in cultivars and mutants used in this study. Asterisks indicate statistically significant differences relative to the original cultivar (*P*<0.01; Dunnett [33]).

**Figure 10:**
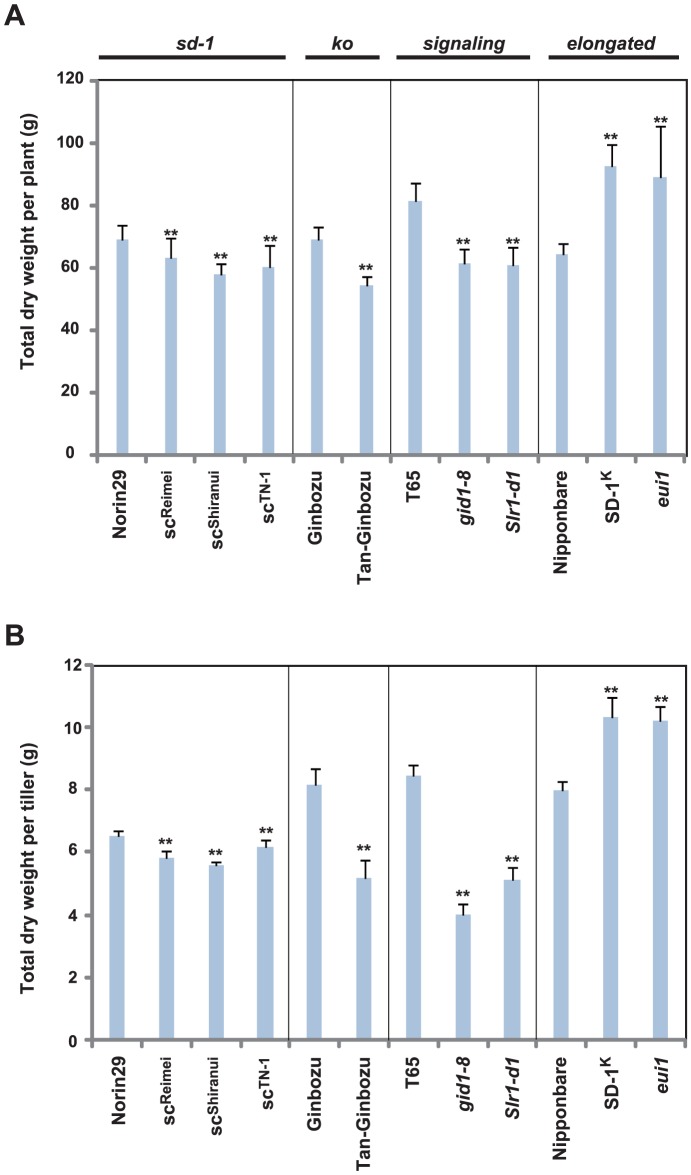
Accumulation of GA leads to increased total biomass in rice. Rice was sampled 40 days after heading, air-dried for 2 weeks, and sampled foliage for dry weight by measuring the foliage. (A) Total dry weight per plant (n = 30), and (B) total dry weight per tiller (n = 10). Asterisks indicate statistically significant differences relative to original cultivars (*P*<0.01; Dunnett [33]).

We also examined the effect of GA on above-ground biomass. First, we measured the number of tillers per plant (Fig. 9). *Tan-Ginbozu*, *gid1-8*, and *Slr1-d1*, which showed intermediate to severe dwarfism showed increased tiller number, whereas the *sd-1* mutants with mild dwarfism showed no difference relative to the original line. The tall mutants also did not show apparent differences relative to their original lines, although there was a decreasing trend observed for *eui1*. Next, we measured the total dry weight per plant. As expected, dwarf and tall GA mutants/line exhibited decreased and increased total biomass, respectively (Fig. 10A). There was also a similar trend for dry weight per tiller (Fig. 10B), although some differences were observed for *Tan-Ginbozu*, *gid1-8*, and *Slr1-d1*. Lines *Tan-Ginbozu*, *gid1-8*, and *Slr1-d1* showed a 36.6, 52.6, and 39.4% reduction in dry weight per tiller compared to their original cultivars, respectively (Fig. 10B), which is a larger reduction than the total dry weight per plant (e.g. 19.4, 16.2, and 16.2% compared to their original cultivars, respectively, Fig. 10A). This is due to increased tiller numbers per plant in these mutants (Fig. 9). Although it increased tiller number, the dwarfism caused by GA deficiency or insensitivity decreased total biomass.

Finally, we examined the grain yield per plant (Figure S1). *sd-1* mutants and Tan-Ginbozu showed slight reduction compared to their original cultivars, whereas, reduction was more severe in *gid1-8* and *Slr1-d1* when compared with T65. For the GA high producing rice, SD-1^K^ and *eui1*, grain yield per plant did not differ to their orginal cultivar Nipponbare.

## Discussion

As mentioned previously, lodging is a serious obstacle in crop production. Lodging disturbs the ripening process, decreases crop yield, and causes poor grain quality [28]. Lodging is typically classified into one of three types based on the underlying cause. The first is the bending-type of lodging, e.g. bending at the middle of the internode without culm breakage. The second is the breaking type of lodging, which is characterized by breaking of internodes in the culm primarily below the third internode. The breaking type of lodging is more serious than the bending-type, because bended culms are still able to transport photosynthetic assimilates from the leaves to the panicles, which is necessary for grain filling. The third type of lodging is the rolling-type. This type of lodging primarily results from failure to develop a deep and widespread root system. In situations where transplants are cultivated (such as rice), this type of lodging is not as serious, since well-developed roots of transplants are generally able to spread deeply into the soil.

In this study, we used two methods to evaluate lodging resistance. One method was assessment of the cLr values, which evaluates the bending type of lodging resistance [18]. This method depends on the traction that a unit length of culm can support, and can be easily measured in the field. We also directly measured the bending load at breaking of culms by using the load-testing machine, Tensilon RTM-25 Orientic, as described by Ookawa and Ishihara [19]. The cLr value of dwarf and semi-dwarf plants was superior to tall plants (Fig. 3). Decreasing plant height lowers the center of gravity and also reduces fresh weight, resulting in decreased “self-weight” moment of the above-ground portion of the plant. This is why dwarf mutants have higher lodging resistance. On the other hand, GA deficiency and insensitivity cause negative pleiotropic effects on rice culms with respect to physical strength, which is determined by their structure and composition. To evaluate structure, we measured the diameter of the 4^th^ internodes, which directly affects the bending load and resistance to the breaking-type lodging. GA-deficient and insensitive plants showed reduced diameter of culm internodes relative to their original lines, whereas tall plants with higher GA levels showed increased diameter (Fig. 4). There was a good correlation between the internode diameter and the plant height, indicating that GA positively regulates the diameter of culm internodes.

GA also seems to positively regulate lignin content (Fig. 5). Namely, plants with lower GA levels (*sd-1* mutants and *Tan-Ginbozu*) or reduced GA signaling (*gid1-8* and *Slr1-d1*) showed reduced lignin content; furthermore, plant with higher GA level (*SD-1^K^*) showed higher lignin content relative to original strains. For the *eui1* plant, although there was a trend of increase in the lignin content compared to Nipponbare, it was not significant. Compared to SD-1, which is responsible for bioactive GA synthesis during the entire growth stage of rice [29], EUI1 has been reported to regulate bioactive GA levels in restricted developmental process; mainly during the heading stage [16]. Such difference might account for the insignificant increase in the lignin content in the *eui1* plant compared to *SD-1^K^*, which should be explored in the future.

Enhanced or suppressed lignification by overproduction of GA biosynthetic or inactivating enzymes, respectively, was also reported in transgenic tobacco [30], which supports our contention that GA is a positive regulator for lignin synthesis. Ookawa and Ishihara [7] examined which cell wall components impact bending stress at the culm by using seven japonica and indica rice cultivars showing different levels of bending stress. Although there was a small difference in the cell wall composition among these cultivars, they found that lignin content of the culm was most significantly correlated with bending stress (r = 0.81) in the seven cultivars. Based on these observations, they concluded that lignin content is the main factor determining varietal differences in bending stress. Thus, GA seems to improve lodging resistance from two different perspectives, e.g. structurally by increasing culm diameter and qualitatively by increasing lignin content. Consequently, the bending moment at breaking, which is the best parameter to evaluate the physical strength of culm, is apparently decreased in most internodes of GA-deficient and insensitive mutants but increased in GA-overproducing mutants (Fig. 8).

We also observed the effect of GA on biomass production. As expected, GA-dependent dwarfism causes significant decreases in the total dry weight of above-ground plant biomass, whereas mutants with elevated GA showed higher biomass (Fig. 10). In rice, biomass is influenced strongly by plant height and tiller number. It is widely accepted that GA induces both cell division and cell elongation and thus GA positively affects plant height [31], [16]. Interestingly, mutants with intermediate and severe dwarfism showed increased numbers of tiller, whereas mutants with mild dwarfism or with taller plant height did not show significant differences in tiller numbers (Fig. 9). In contrast to findings with strigolactone and cytokinin, the relationship between GA and tiller number has not been well-investigated. However, there is a report that transgenic rice overproducing the GA-inactivating enzyme, GA 2 oxidase, exhibited increased tillering by negatively regulating expression of *Os TEOSINTE BRANCHED1* (*OsTB1*), a positive regulator for the strigolactone signaling [32]. Our results agree with this report; however, the impact of GA on tiller number is much less than its effect on plant height.

Taken collectively, our results demonstrate that GA has both positive and negative effects on lodging resistance, and positive effect on biomass production (Table 1). GA deficiency or insensitivity induces dwarfism, which decreases the “self-weight” moment of above-ground parts of plants, resulting in improved lodging resistance. In terms of culm strength, GA deficiency or insensitivity reduces culm diameter and decreases lignin content, both of which decrease lodging resistance. The dwarfism caused by GA deficiency or insensitivity has a negative effect on biomass production and increased tiller numbers do not fully compensate for reduced biomass. Thus, strategies to increase crop productivity using GA-deficient dwarf plants may have an intrinsic paradox. Notably, the advances in increasing rice production have been small after the huge success of the Green Revolution, and the demerits of short plant stature have been discussed from a crop science perspective [5]. Furthermore, the increasing demand for large biomass for the production of biofuels has led to a paradigm shift for alternative plant architecture for bioenergy crops. Our results clearly demonstrate that increased GA levels promotes taller, wider plants, which both contribute to increased biomass. Thus, alternative strategies using tall plants with higher GA levels should be explored for developing new cultivars with high grain and high biomass, concomitantly with the implementation of novel genes that enhance culm strength and grain yield.

**Table 1:** Summary of morphological traits, lodging resistance and biomass production of GA mutants used in this study.

	lodging resistance						
	culm length	bending-type	breaking-type	culm diameter	lignin content	tiller number	biomass
*sd-1* mutants	−a	+^b^	−	−	−	no effect	−
*Tan-Ginbozu*	−	+	−	−	−	+	−
*gid1-8*, *Slr1-d1*	−	+	−	−	−	+	−
SD-1^K^, *eui1*	+	−	+	+	+	no effect	+

a Those that decreased compared to their original cultivar.

b Those that increased compared to their original cultivar.

## Supporting Information

Figure S1 **Grain yield per plant of rice used in this study.** Rice was sampled 40 days after heading, air-dried for 2 weeks, and total grain yield per plant were measured (n = 30). Asterisks indicate statistically significant differences relative to original cultivars (*P<0.05, **P<0.01; Dunnett [33]).
(EPS)
